# Long non-coding RNA FER1L4 promotes osteogenic differentiation of human periodontal ligament stromal cells via miR-874-3p and vascular endothelial growth factor A

**DOI:** 10.1186/s13287-019-1519-z

**Published:** 2020-01-03

**Authors:** Yiping Huang, Yineng Han, Runzhi Guo, Hao Liu, Xiaobei Li, Lingfei Jia, Yunfei Zheng, Weiran Li

**Affiliations:** 1 Department of Orthodontics, Peking University School and Hospital of Stomatology, 22 Zhongguancun South Avenue, Haidian District, Beijing, 100081 China; 2 Central Laboratory, Peking University School and Hospital of Stomatology, Beijing, 100081 China; 3 National Engineering Laboratory for Digital and Material Technology of Stomatology, Beijing Key Laboratory of Digital Stomatology, Beijing, 100081 China

**Keywords:** Long non-coding RNA, Periodontal ligament stromal cells, FER1L4, Osteogenesis, miR-874-3p, VEGFA

## Abstract

**Background:**

Periodontal ligament stromal cells (PDLSCs) are ideal cell sources for periodontal tissue repair and regeneration, but little is known about what determines their osteogenic capacity. Long non-coding RNAs (lncRNAs) are important regulatory molecules at both transcriptional and post-transcriptional levels. However, their roles in the osteogenic differentiation of PDLSCs are still largely unknown.

**Methods:**

The expression of lncRNA Fer-1-like family member 4 (FER1L4) during the osteogenic differentiation of PDLSCs was detected by quantitative reverse transcription polymerase chain reaction. Overexpression or knockdown of FER1L4 was used to confirm its regulation of osteogenesis in PDLSCs. Alkaline phosphatase and Alizarin red S staining were used to detect mineral deposition. Dual luciferase reporter assays were used to analyze the binding of miR-874-3p to FER1L4 and vascular endothelial growth factor A (VEGFA). Bone regeneration in critical-sized calvarial defects was assessed in nude mice. New bone formation was analyzed by micro-CT, hematoxylin and eosin staining, Masson’s trichrome staining, and immunohistochemical analyses.

**Results:**

FER1L4 levels increased gradually during consecutive osteogenic induction of PDLSCs. Overexpression of FER1L4 promoted the osteogenic differentiation of PDLSCs, as revealed by alkaline phosphatase activity, Alizarin red S staining, and the expression of osteogenic markers, whereas FER1L4 knockdown inhibited these processes. Subsequently, we identified a predicted binding site for miR-874-3p on FER1L4 and confirmed a direct interaction between them. Wild-type FER1L4 reporter activity was significantly inhibited by miR-874-3p, whereas mutant FER1L4 reporter was not affected. MiR-874-3p inhibited osteogenic differentiation and reversed the promotion of osteogenesis in PDLSCs by FER1L4. Moreover, miR-874-3p targeted VEGFA, a crucial gene in osteogenic differentiation, whereas FER1L4 upregulated the expression of VEGFA. In vivo, overexpression of FER1L4 led to more bone formation compared to the control group, as demonstrated by micro-CT and the histologic analyses.

**Conclusion:**

FER1L4 positively regulates the osteogenic differentiation of PDLSCs via miR-874-3p and VEGFA. Our study provides a promising target for enhancing the osteogenic potential of PDLSCs and periodontal regeneration.

## Introduction

Periodontitis is one of the most common oral diseases, leading to the destruction of the tissues supporting the teeth, including the periodontal ligament, cementum, and alveolar bone [1]. Effective reconstruction of periodontal supporting structures is the ultimate objective of periodontal treatment [2]. The periodontal ligament is a soft tissue located in the alveolar socket that maintains dental homeostasis, nutrition, and alveolar bone remodeling [3, 4]. Periodontal ligament stromal cells (PDLSCs), which are derived from the periodontal ligament tissue, are heterogeneous, nonclonal cultures of stromal cells consisting of stem cells with different multipotential properties, committed progenitors, and differentiated cells [5]. They can form new bone, new cementum, and functional periodontal ligament and thus are considered as ideal cell sources for periodontal tissue repair and regeneration [6, 7]. However, the osteogenic capacity of PDLSCs is greatly inhibited by many factors, such as aging [8], inflammation [9], and hypoxia [10]. Therefore, further investigation of the osteogenic regulation of PDLSCs would be helpful to improve their osteogenic potential and periodontal regeneration.

Non-coding RNAs (ncRNAs) are a group of RNAs that have no protein-coding properties but serve as important regulatory transcripts in biological control and pathology [11]. A class of small ncRNAs, including miR-23a [12], miR-24-3p [13], miR-214 [14], and miR-543 [15], participates in the regulation of osteogenic differentiation in PDLSCs. Long non-coding RNAs (lncRNAs), another important subset of ncRNAs defined as ncRNAs > 200 nucleotides (nt) in length [16, 17], have gradually attracted attention in the field of cell differentiation. However, only a few lncRNAs, including ANCR [18], lncPCAT1 [19], TUG1 [20], and MEG3 [21], are demonstrated to participate in the osteogenic differentiation of PDLSCs. Their roles are still largely unknown in this field. It is recently recognized that lncRNAs and miRNAs suppress each other and form a precise regulatory network [22, 23]. miRNAs regulate gene expression by directly binding to the 3′-untranslated regions (3′-UTRs) of messenger RNAs (mRNAs) [24], while lncRNAs acquire functionality by acting as a sponge for miRNAs to regulate the abundant target genes of miRNAs [25]. The regulatory networks composed of lncRNAs and miRNAs play important roles in various cellular processes [22, 23].

Fer-1-like family member 4 (FER1L4), a newly discovered lncRNA, is located on chromosome 20 in humans. It does not encode a protein but rather a 6717-bp ncRNA that is highly expressed in embryonic and adult tissues. FER1L4 has been studied in several kinds of cancer, including colon cancer [26], gastric carcinoma [27], hepatocellular carcinoma [28, 29], osteosarcoma [30], and gliomas [31]. However, no study had considered the role and action of FER1L4 in osteogenic differentiation. It remains unclear whether it is important for stromal cell differentiation, and if so, what is its mechanism of action? Therefore, in this study, we investigated the role and mechanism of FER1L4 in the osteogenic differentiation of PDLSCs.

## Materials and methods

### Cell culture

This study was approved by the Ethics Committee of Peking University School of Stomatology (PKUSSIRB-2011007). Human PDLSCs were isolated and characterized as described previously [32]. Briefly, periodontal tissues were scraped from the middle third of the root of healthy premolars from three donors who underwent orthodontic extraction (one male and two females, aged 12–18 years). The tissues were cut and digested in equal volumes of type I collagenase and dispase for 1 h at 37 °C. Then, the isolated cells were cultured in α-modified Eagle’s medium supplemented with 10% fetal bovine serum and 1% penicillin/streptomycin. After 7 days, the stromal cells began growing out from the tissue. They were passaged and further expanded until passage 4. The cell surface markers and multipotency were characterized as described previously [32]. The osteogenic differentiation of PDLSCs was induced once cells reached 70–80% confluence using standard medium supplemented with 100 nM dexamethasone, 200 μM l-ascorbic acid, and 10 mM β-glycerophosphate (Sigma-Aldrich, St. Louis, MO, USA). Human embryonic kidney (293T) cells were obtained from the American Type Culture Collection (Manassas, VA, USA) and cultured in Dulbecco’s modified Eagle’s medium supplemented with 10% fetal bovine serum and 1% penicillin/streptomycin.

### Generation of constructs

Full-length FER1L4 (Gene Bank accession number, NR_119376.1) cDNA was cloned into the pQLL vector (Qinglan Biotech. Co., Wuxi, China) and named pQLL-FER1L4 (Additional file 1: Figure S1A). Putative miR-874-3p binding sites in FER1L4 and vascular endothelial growth factor A (VEGFA), both wild-type (WT) and mutant (MU), were synthesized and cloned downstream of the luciferase gene in luciferase vectors (GenePharma Co., Shanghai, China).

### RNA oligoribonucleotides

RNA oligoribonucleotides including miR-874-3p mimic, small-interfering RNAs (siRNAs) targeting FER1L4, and their corresponding miRNA control (miR-NC) and siRNA control (siNC) were obtained from GenePharma Co. The sequences are listed in Additional file 1: Table S1.

### Transient transfection

Cells were plated in 6-well plates before transfection. After reaching 80% confluence, they were transfected with 2 μg plasmid or 100 nM miRNA mimic or siRNAs using Lipofectamine 3000 (Invitrogen, Carlsbad, CA, USA) according to the manufacturer’s procedure. One well was transfected with a vector expressing green fluorescent protein to assess the transfection efficiency.

### Alkaline phosphatase staining and activity

Alkaline phosphatase (ALP) staining was performed according to the protocol provided with the NBT/BCIP staining kit (CoWin Biotech, Beijing, China). Briefly, after osteogenic induction for 7 days, the cultured cells were fixed in 4% paraformaldehyde and then incubated in alkaline solution for 20 min. ALP activity was analyzed using a colorimetric assay kit (Biovision, Milpitas, CA, USA). Cultured cells were washed with PBS, lysed with 1% Triton X-100 (Sigma-Aldrich), scraped into distilled water, and subjected to 3 cycles of freezing and thawing. ALP activity was determined at 405 nm using p-nitro-phenyl phosphate as a substrate. The total protein content was measured using the bicinchoninic acid (BCA) method with a Pierce protein assay kit (Thermo Fisher Scientific, Waltham, MA, USA). ALP activity was calculated after normalization to protein content.

### Alizarin red S staining and quantification

Alizarin red S staining and quantification were performed as described previously [33]. After osteogenic induction for 14 days, cultured cells were fixed in 4% paraformaldehyde and then stained with 0.1% Alizarin red S (pH 4.2; Sigma-Aldrich). For quantitative assessment of the degree of mineralization, the stain was dissolved in cetylpyridinium chloride (Sigma-Aldrich) and the absorbance at 570 nm was measured. The final value was calculated after normalization to protein content.

### RNA isolation and quantitative reverse transcription polymerase chain reaction

Total RNA was extracted using TRIzol reagent (Invitrogen) according to the manufacturer’s procedure and reverse-transcribed into cDNA using a cDNA Reverse Transcription Kit (Takara, Tokyo, Japan). Quantitative reverse transcription polymerase chain reaction (qRT-PCR) was performed on 1 μg RNA using SYBR Green PCR Master Mix on an ABI Prism 7500 Real-Time PCR System (Applied Biosystems, Foster City, CA, USA). The following settings were used: 95 °C for 10 min followed by 40 cycles of 95 °C for 15 s and 60 °C for 1 min. miR-874-3p was reverse-transcribed using a specific RT primer (RiboBio, Guangzhou, China) according to the manufacturer’s protocol. Glyceraldehyde 3-phosphate dehydrogenase (GAPDH) was used as an endogenous normalization control for mRNAs and lncRNAs. U6 was used as an endogenous normalization control for miRNAs. The primers used for FER1L4, ALP, osteocalcin (OCN), runt-related transcription factor 2 (RUNX2), and VEGFA are listed in Additional file 1: Table S1. Data were analyzed using the 2^−ΔΔCt^ relative expression method as described previously [33].

### Western blot

Western blot was performed as described previously [33]. Briefly, cells were harvested, washed, and lysed in radioimmunoprecipitation assay buffer. Protein content was determined using the BCA method. Proteins were separated by 12% sodium dodecyl sulfate-polyacrylamide gel electrophoresis and electroblotted onto the polyvinylidene fluoride membranes. After blocking, proteins were detected by overnight incubation with primary antibodies against OCN (Abcam, Cambridge, UK), RUNX2 (Abcam), VEGFA (Abcam), and GAPDH (Zhongshan Goldenbridge, Beijing, China) at 1:1000 dilution. After washing, the membranes were incubated with secondary antibodies (Zhongshan Goldenbridge; 1:10,000 dilution) at room temperature for 1 h. Specific complexes were visualized using an enhanced chemiluminescence kit (Applygen, Beijing, China). Band intensities were quantified using ImageJ software (http://rsb.info.nih.gov/ij/). The background was subtracted, and the signal of each target band was normalized to that of GAPDH.

### Dual-luciferase reporter assay

Luciferase assays were performed as described previously [33]. Briefly, cells grown in 48-well plates were transfected with 40 ng luciferase reporter, 4 ng *Renilla*, and 100 nM miR-874-3p mimic or control. Firefly and *Renilla* luciferase activities were measured 24 h after transfection using the Dual-Luciferase Reporter Assay System (Promega, Beijing, China). The light intensity from the firefly luciferase was normalized to *Renilla* luciferase.

### In vivo bone formation assay

Animal experiments were approved by the Peking University Animal Care and Use Committee (LA2018305). PDLSCs transfected with pQLL-NC or pQLL-FER1L4 were cultured with osteogenic induction for 1 week before the in vivo study. The cells were seeded in polylactic-co-glycolic acid (PLGA; Melone, Dalian, China) scaffolds prepared as thin circular slices (*Φ* = 4 mm). Nude mice ~ 5 weeks old were purchased from Vital River Laboratory Animal Technology Co. (Beijing, China) and randomly divided into two groups (five mice/group). The critical-sized calvarial defect mouse model was constructed under general anesthesia as described previously [34]. After removing the pericranium, critical-sized calvarial defects (*Φ* = 4 mm) were made using a dental drill. Then, the seeded scaffolds were gently transplanted into the defects, and the skin incision was closed with 5-0 Vicryl sutures. After 8 weeks, the skulls were harvested and fixed in 4% paraformaldehyde.

### Micro-computed tomography analyses

Micro-computed tomography (micro-CT) analyses of specimens were conducted using a high-resolution Inveon Micro-CT (Siemens, Munich, Germany). Images were acquired at an effective pixel size of 8.99 μm at 80 kV, 500 μA, and with a 1500-ms exposure time. The samples were placed in one container and scanned with uniform parameters. Three-dimensional reconstruction was performed using the Inveon Research Workplace 3.0 software (Siemens).

### Hematoxylin and eosin staining, Masson’s trichrome staining, and immunohistochemical analyses

Samples were decalcified in 10% ethylene diamine tetra-acetic acid (pH 7.4) for 1 month, then washed, dehydrated, and embedded in paraffin. The sections were cut at 7 μm thickness and stained with hematoxylin and eosin (H&E) and Masson’s trichrome. The sections were also evaluated by immunohistochemistry, as described previously [33]. Briefly, the sections were blocked with 3% goat serum albumin (Zhongshan Goldenbridge) and then incubated with primary antibody against OCN (Abcam) overnight at 4 °C. Then, they were incubated with the corresponding secondary antibodies and processed using a detection kit (Zhongshan Goldenbridge). Images were captured on a BX51 light microscope equipped with a DP70 camera (Olympus, Co., Tokyo, Japan).

### Statistical analyses

Statistical analyses were performed using SPSS version 16.0 (SPSS, Chicago, IL, USA). All data are expressed as the mean ± SD of at least three independent experiments. Differences between the two groups were analyzed using Student’s *t* test. In cases of multiple group testing, a one-way analysis of variance was used. Pearson’s correlation coefficient was used to analyze the correlation between the two variables. A two-tailed value of *p* < 0.05 was considered statistically significant.

## Results

### Expression of lncRNA FER1L4 is significantly upregulated during osteogenic differentiation of PDLSCs

**Fig. 1:**
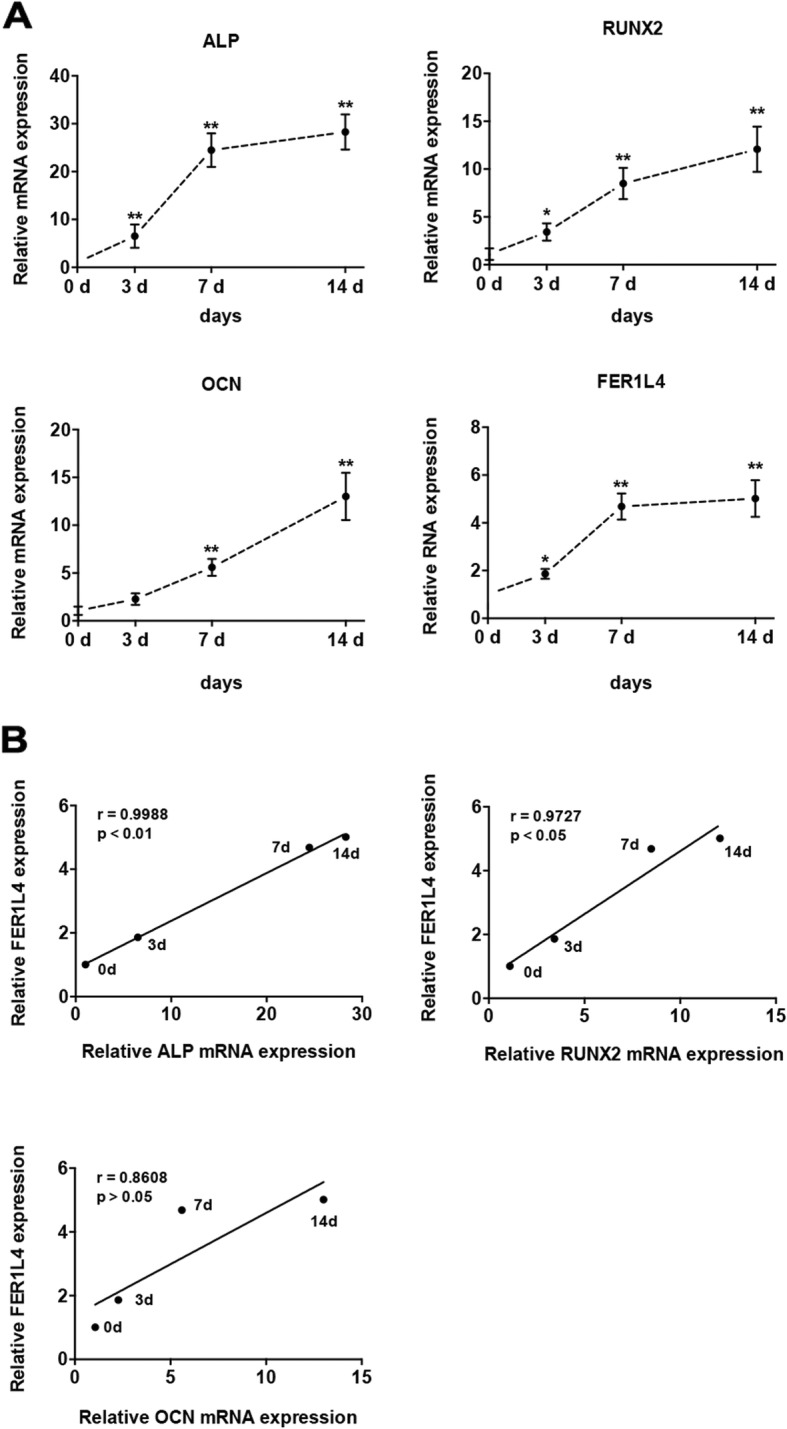
Dynamic expression profile of FER1L4 during the osteogenic differentiation of PDLSCs. **a** Relative expression of FER1L4 and the osteogenic markers ALP, RUNX2, and OCN at 0, 3, 7, and 14 days of osteogenic differentiation. **b** Correlation analyses were performed between FER1L4 levels and ALP, RUNX2, and OCN mRNA levels during osteogenic differentiation. Results are presented as mean ± SD (*p < 0.05, **p < 0.01)

After the osteogenic induction of PDLSCs, the mRNA expression of the three osteogenic markers ALP, RUNX2, and OCN was significantly increased, indicating the successful induction of PDLSCs into the osteogenic lineage. Next, the dynamic expression profile of FER1L4 were assessed at 0, 3, 7, and 14 days of induction. Its expression was significantly upregulated, reaching > 5-fold of the initial level on day 14 (Fig. 1a). There were also positive correlations between the levels of FER1L4 and the osteogenic genes ALP and RUNX2 (Fig. 1b), indicating that lncRNA FER1L4 is involved in osteogenesis.

### FER1L4 promotes osteogenic differentiation of PDLSCs

To determine the function of FER1L4 in the osteogenesis of PDLSCs, pQLL-FER1L4 vector was used to overexpress and siRNA targeting FER1L4 (siFER1L4) was used to knock down FER1L4 expression. The transfection efficiency was > 70% (Additional file 1: Figure S1B). The transfection effects were further confirmed by qRT-PCR. FER1L4 expression was increased > 5000-fold in the overexpression group and decreased ~ 60% in the knockdown group (Additional file 1: Figure S1C). Then, the vectors or siRNAs were transfected into PDLSCs in growth media, and the media were changed to osteogenic media. On day 3, the PDLSCs were transfected again with vectors or siRNAs and then harvested on day 7 or day 14.

**Fig. 2:**
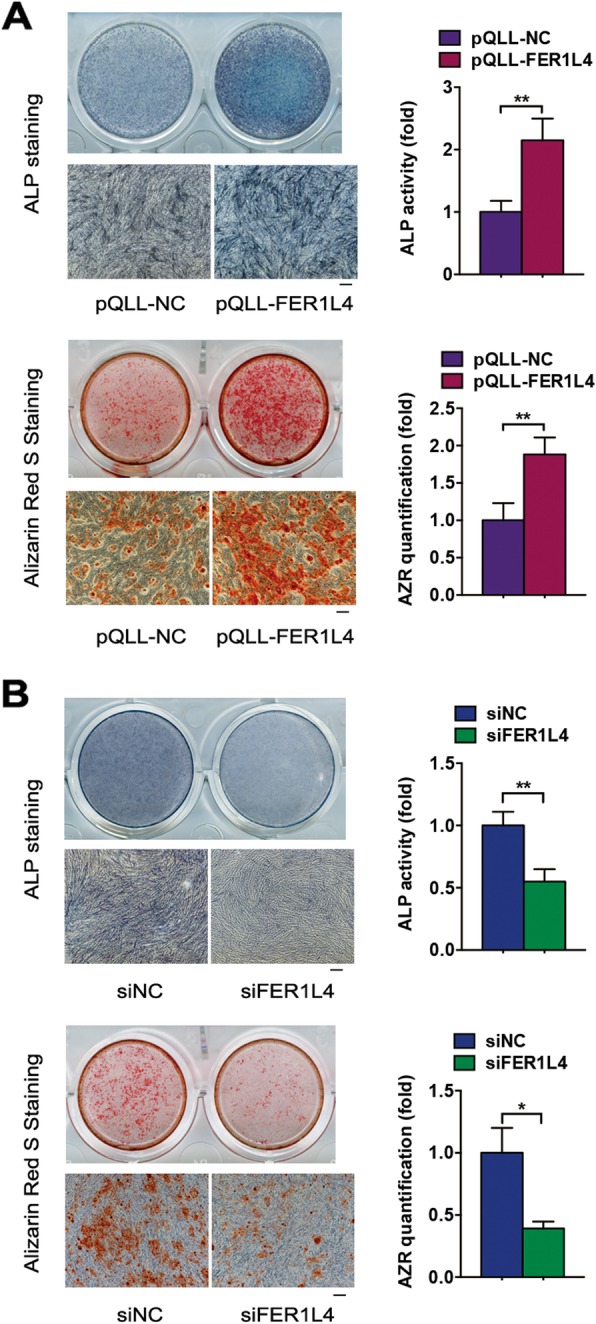
FER1L4 promoted the osteogenic differentiation of PDLSCs. Cells were transfected with a vector expressing FER1L4 (pQLL-FER1L4), siRNA targeting FER1L4 (siFER1L4), or their controls. **a** Images of ALP staining on day 7 of osteogenic differentiation and Alizarin red S staining on day 14 of osteogenic differentiation in the pQLL-NC and pQLL-FER1L4 groups. Scale bar, 100 μm. Histograms show ALP activity and quantification of Alizarin red S staining by spectrophotometry. **b** Images of ALP staining and Alizarin red S staining in the siNC and siFER1L4 groups. Scale bar, 100 μm. Histograms show ALP activity and quantification of Alizarin red S staining by spectrophotometry. Results are presented as mean ± SD (*p < 0.05, **p < 0.01)

After 7 days of induction, ALP staining and activity were increased by FER1L4 overexpression and decreased by FER1L4 knockdown (Fig. 2a, b). After 14 days of induction, matrix mineralization, as revealed by Alizarin red S staining, was enhanced in the FER1L4 overexpression group and reduced in the FER1L4 knockdown group (Fig. 2a, b).

**Fig. 3:**
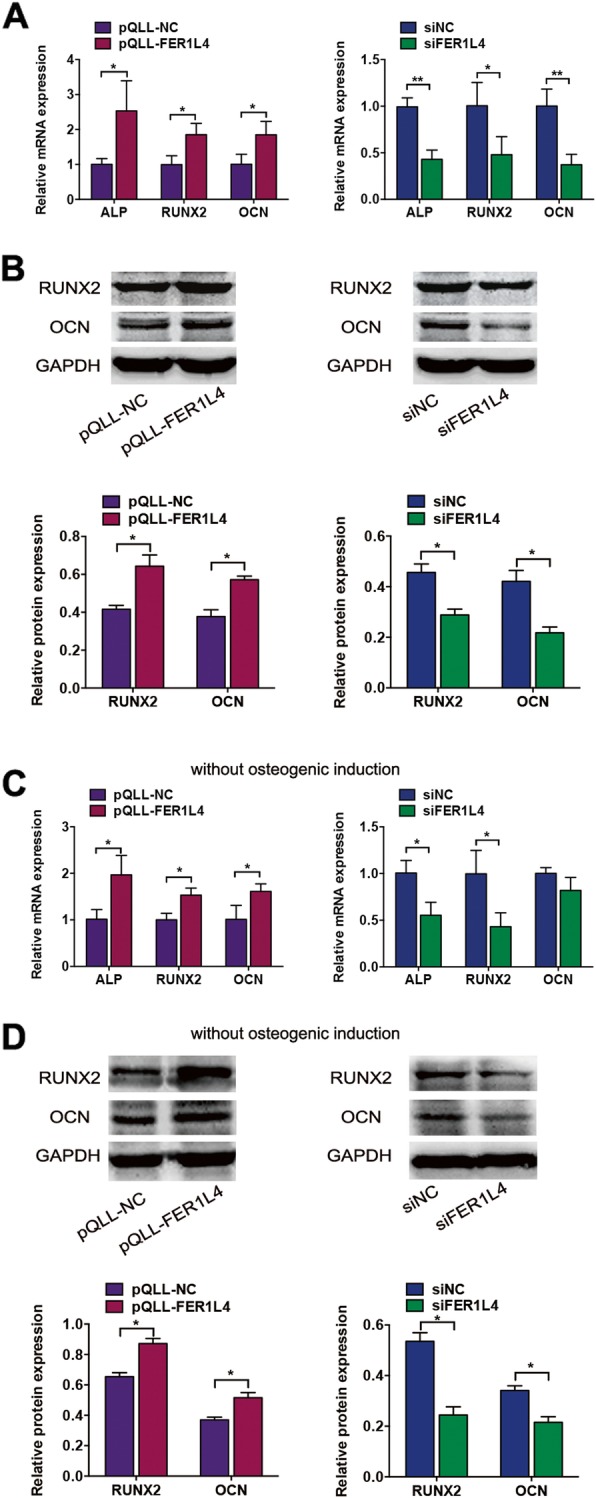
FER1L4 upregulated the expression of osteogenic markers in PDLSCs. Cells were transfected with a vector expressing FER1L4 (pQLL-FER1L4), siRNA targeting FER1L4 (siFER1L4), or their controls. **a** Relative mRNA expression of the osteogenic markers ALP, RUNX2, and OCN on day 7 of osteogenic induction. **b** Western blot analyses of RUNX2 and OCN on day 7 of osteogenic induction. Histograms show quantification of the band intensities. **c** Relative mRNA expression of ALP, RUNX2, and OCN without osteogenic induction. **d** Western blot analyses of RUNX2 and OCN without osteogenic induction. Histograms show quantification of the band intensities. Results are presented as mean ± SD (*p < 0.05, **p < 0.01)

The expression of osteogenic markers was assessed 7 days after osteogenic induction in transfected PDLSCs. FER1L4 overexpression significantly upregulated the mRNA levels of ALP, RUNX2, and OCN, whereas FER1L4 knockdown significantly downregulated the expression of these genes (Fig. 3a). The regulation of RUNX2 and OCN by FER1L4 was confirmed at the protein level (Fig. 3b). Even when the cells were cultured in medium without osteogenic supplements for 3 days, the mRNA expression of ALP, RUNX2, and OCN was upregulated in cells overexpressing FER1L4 and downregulated in FER1L4-knockdown cells (Fig. 3c). The protein expression of RUNX2 and OCN showed similar trends (Fig. 3d).

### FER1L4 acts as a sponge of miR-874-3p

**Fig. 4:**
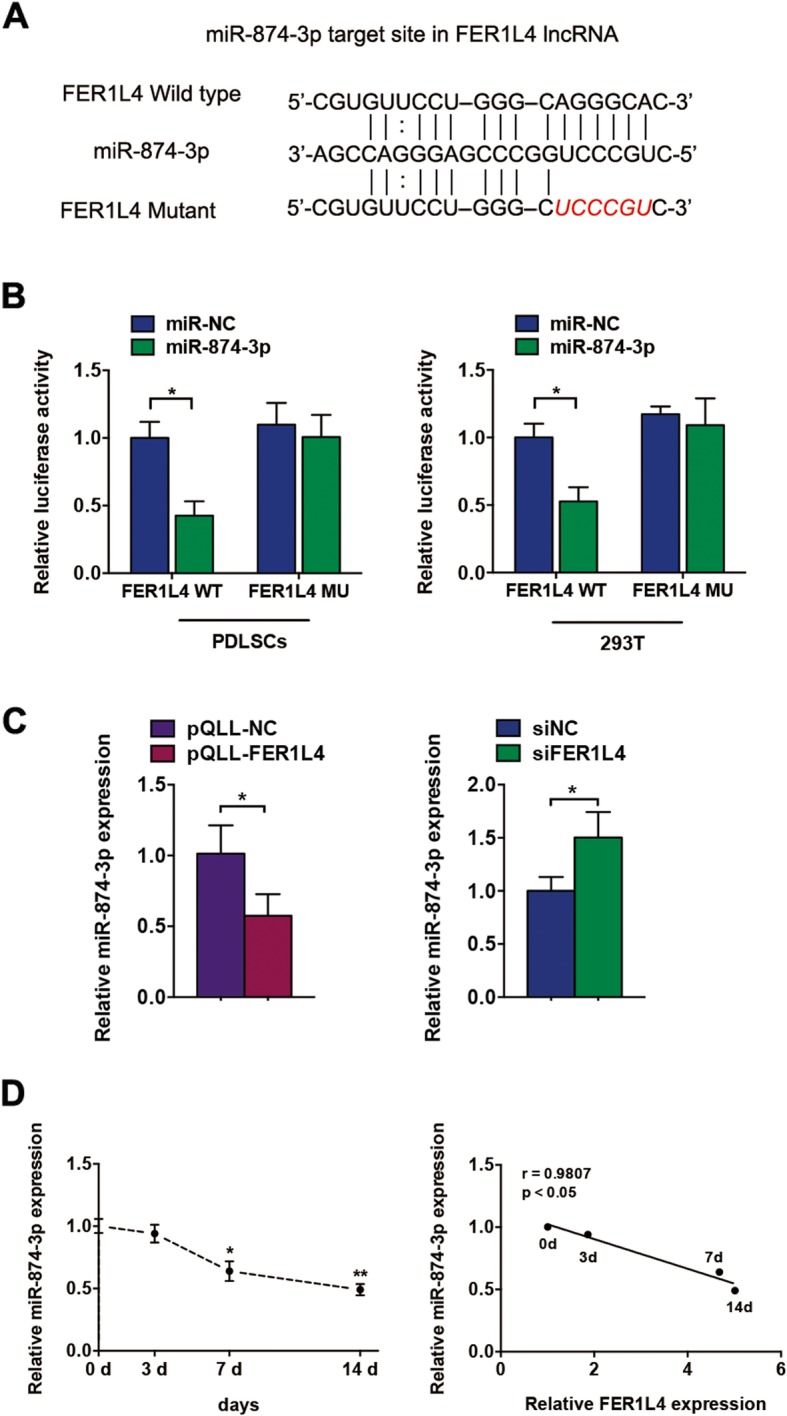
FER1L4 acted as a sponge of miR-874-3p. **a** Schematic diagram of the miR-874-3p putative binding site and mutant site in FER1L4. **b** The relative luciferase activities of luciferase reporters containing WT or MU FER1L4 transcripts in PDLSCs and 293T cells transfected with miR-874-3p. **c** The levels of miR-874-3p in PDLSCs with overexpressed or knocked down FER1L4. **d** The expression profile of miR-874-3p in PDLSCs at 0, 3, 7, and 14 days of osteogenic induction and the correlation analyses between miR-874-3p levels and FER1L4 levels during osteogenic induction. Results are presented as mean ± SD (*p < 0.05, **p < 0.01)

To determine whether FER1L4 acts as an miRNA sponge that competes with mRNA for binding to miRNAs, we identified a potential binding site for miR-874-3p on FER1L4 (Fig. 4a) based on starBase (http://starbase.sysu.edu.cn). Then, luciferase reporters carrying the WT or MU target site on FER1L4 were constructed. FER1L4-WT reporter activity was significantly inhibited by miR-874-3p in PDLSCs and 293T cells, whereas the FER1L4-MU reporter was not affected (Fig. 4b), suggesting that miR-874-3p directly binds to the FER1L4 target site. miR-874-3p levels also decreased after FER1L4 overexpression and increased following FER1L4 knockdown in PDLSCs (Fig. 4c). Furthermore, there was a gradual decrease of miR-874-3p expression in PDLSCs during osteogenic differentiation, and there was a negative correlation between miR-874-3p expression and FER1L4 expression during osteogenesis (Fig. 4d).

### miR-874-3p inhibits osteogenic differentiation of PDLSCs

**Fig. 5:**
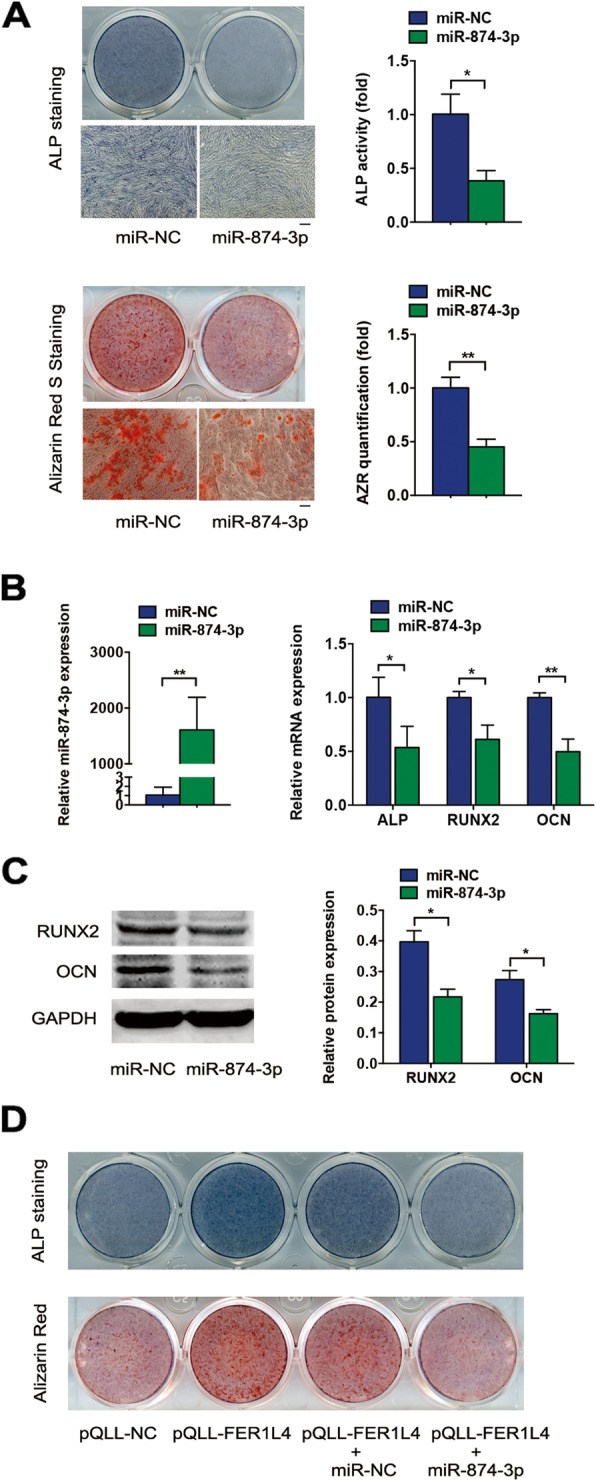
miR-874-3p inhibited the osteogenic differentiation of PDLSCs and reversed the effects of FER1L4. **a** Images of ALP staining on day 7 of osteogenic differentiation and Alizarin red S staining on day 14 of osteogenic differentiation in PDLSCs transfected with miR-NC or miR-874-3p mimic. Scale bar, 100 μm. Histograms show ALP activity and quantification of Alizarin red S staining by spectrophotometry. **b** Transfection efficiency of miR-874-3p overexpression in PDLSCs and relative mRNA expression of ALP, RUNX2, and OCN on day 7 of osteogenic induction. **c** Western blot analyses of RUNX2 and OCN on day 7 of osteogenic induction in PDLSCs transfected with miR-NC or miR-874-3p mimic. Histograms show quantification of the band intensities. **d** Images of ALP staining and Alizarin red S staining in PDLSCs co-transfected with pQLL-FER1L4 with or without miR-874-3p. Results are presented as mean ± SD (*p < 0.05, **p < 0.01)

To determine the effects of miR-874-3p on the osteogenesis of PDLSCs, miR-874-3p mimic was transfected into the cells. ALP staining and activity decreased significantly after 7 days of osteogenic induction, and Alizarin red S staining decreased after 14 days of induction (Fig. 5a). miR-874-3p was overexpressed > 1000-fold after transfection (Fig. 5b). The miR-874-3p mimic downregulated the mRNA expression of ALP, RUNX2, and OCN (Fig. 5b) and the protein expression of RUNX2 and OCN (Fig. 5c). Given that FER1L4 acts as a sponge of miR-874-3p, FER1L4 and miR-874-3p were co-transfected into PDLSCs. miR-874-3p partially reversed the ability of FER1L4 to promote osteogenic differentiation, as revealed by ALP and Alizarin red S staining (Fig. 5d).

### VEGFA is a target of FER1L4/miR-874-3p

**Fig. 6:**
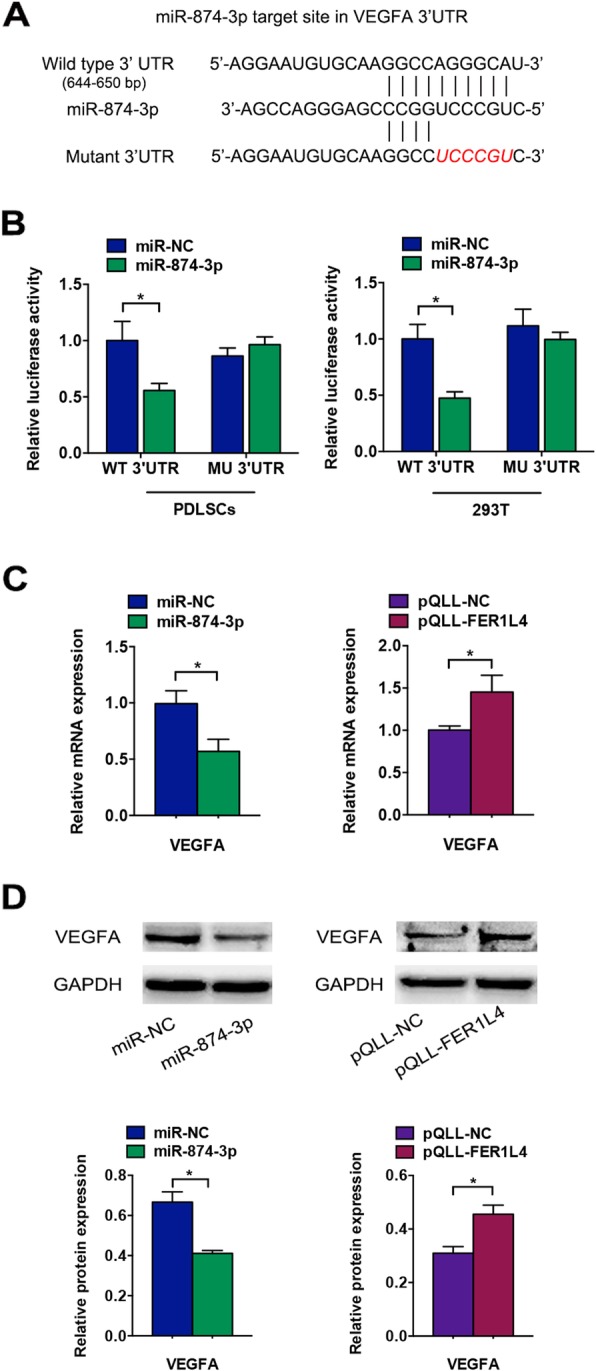
VEGFA was a target of the FER1L4/miR-874-3p regulatory network during osteogenesis. **a** Schematic diagram of the miR-874-3p putative binding site in WT and MU VEGFA 3′-UTR. **b** The relative luciferase activities of luciferase reporters containing WT or MU VEGFA 3′-UTR in PDLSCs and 293T cells transfected with miR-874-3p. **c** Relative mRNA expression of VEGFA in PDLSCs overexpressing miR-874-3p or FER1L4. **d** Western blot analyses of VEGFA in PDLSCs overexpressing miR-874-3p or FER1L4. Histograms show quantification of the band intensities. Results are presented as mean ± SD (*p < 0.05)

The target prediction algorithms TargetScan (http://www.targetscan.org) and starBase (http://starbase.sysu.edu.cn) were used to predict potential targets of miR-874-3p. Both showed that the 3′-UTR of VEGFA contained a miR-874-3p binding site (Fig. 6a). VEGFA is known to be involved in osteogenesis. Thus, based on the predictions, luciferase reporter constructs carrying WT or MU VEGFA 3′-UTR were generated. Reporter activity was reduced ~ 50% by miR-874-3p in PDLSCs and 293T cells, and mutation of the target site relieved this reduction (Fig. 6b). The mRNA and protein expression of VEGFA was decreased in PDLSCs transfected with miR-874-3p mimic (Fig. 6c, d). Meanwhile, the overexpression of FER1L4 increased the mRNA and protein levels of VEGFA (Fig. 6c, d), confirming that FER1L4 regulates VEGFA.

### FER1L4 promotes bone formation in vivo

**Fig. 7:**
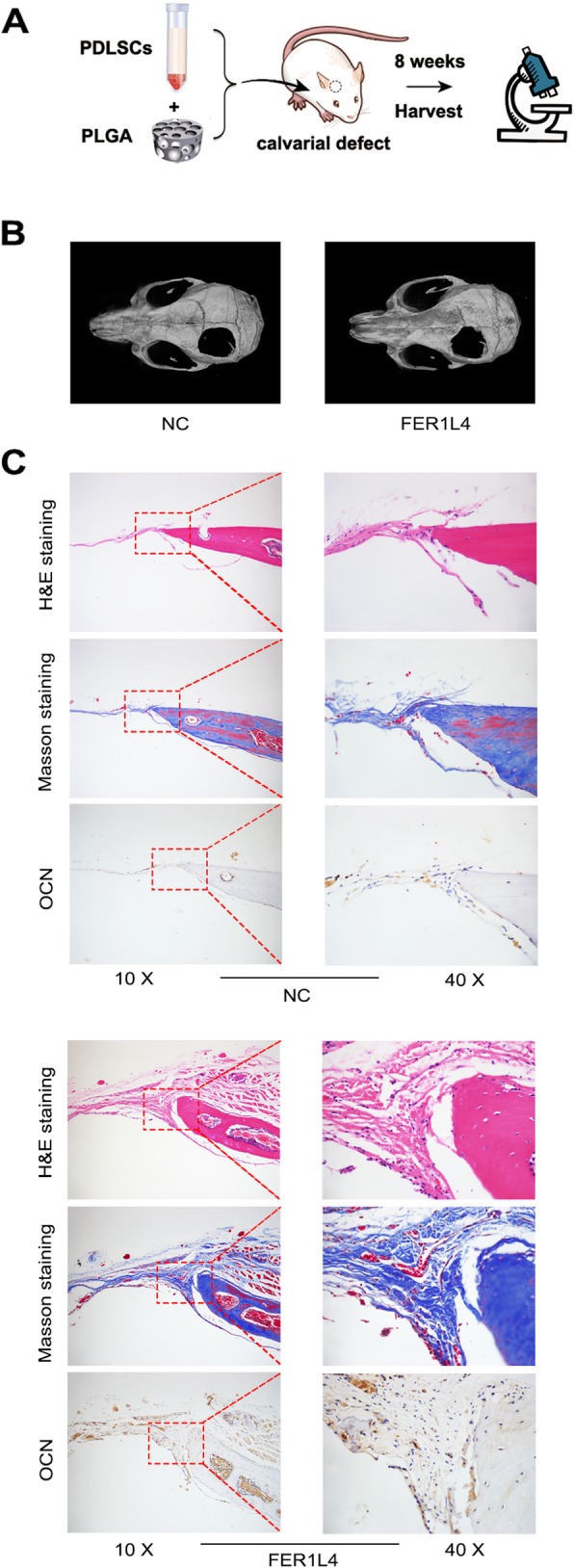
FER1L4 increased bone formation in critical-sized calvarial defects in mice. Mice were transplanted with PLGA scaffolds combined with cultured PDLSCs overexpressing FER1L4 or not. **a** Schematic diagram illustrating the experimental setup. **b** Micro-CT images of bone formation in each group. **c** H&E staining, Masson’s trichrome staining, and immunohistochemical staining of OCN in each group

To further confirm the role of FER1L4 in the osteogenesis of PDLSCs, in vivo animal experiments were conducted. PDLSCs with or without FER1L4 overexpression were loaded on PLGA scaffolds and implanted in calvarial defects of nude mice. After 8 weeks, samples were harvested (Fig. 7a). Three-dimensional reconstructed images showed more new bone formation in the FER1L4-overexpressing group (Fig. 7b). Histological observations were consistent with the results of micro-CT analyses. Bone tissue in H&E staining and collagen organization, as evidenced by a blue color in Masson’s trichrome staining, had formed around the defects in both groups. The amount of new collagen fibers and bone tissue was significantly higher in the FER1L4-overexpressing group (Fig. 7c). Consistent with this, immunohistochemical staining showed that the size and intensity of positive staining for OCN were higher in the FER1L4 group (Fig. 7c).

## Discussion

We demonstrated that FER1L4 levels increased during osteogenic induction. Overexpression of FER1L4 promoted osteogenesis whereas knockdown decreased the osteogenic potential of PDLSCs both in vivo and in vitro. Although substantial evidence shows that lncRNAs are involved in various cellular processes and biological control, only a few have been reported to regulate the osteogenic differentiation of PDLSCs: ANCR [18], lncPCAT1 [19], TUG1 [20], and MEG3 [21]. FER1L4 is an abundant and conserved long non-coding transcript. Previous studies have reported an anti-tumorigenic role of FER1L4 in several kinds of cancer [26–30]. FER1L4 expression is downregulated in hepatocellular carcinoma and attenuates cell proliferation, migration, and invasion by blocking the PI3K/AKT pathway [28]. In addition, it modulates PTEN expression to suppress tumorigenesis [30]. However, no study has considered the role and action of FER1L4 on osteogenic differentiation. Here, we demonstrated its positive regulatory effects on the osteogenesis of PDLSCs. Many efforts have been made to improve the osteogenic potential of PDLSCs. In a preliminary study, insulin-like growth factor binding protein 5 was injected locally to promote periodontal tissue regeneration in a mini-pig model of periodontitis [35]. Our findings on the effects of FER1L4 may provide a new target to promote periodontal repair and regeneration.

Regarding the sponge effects of lncRNAs, we investigated the underlying interaction between FER1L4 and miRNA. FER1L4 served as a competitive endogenous RNA to bind to miR-874-3p. miR-874-3p is involved in the viability [36], apoptosis [37], and chemoresistance of cancer cells [38]. miR-874 is also involved in osteoblast proliferation and differentiation in osteoporotic rats through the Hedgehog signaling pathway [39]. A recent study identified 116 differentially expressed miRNAs during the osteogenic differentiation of PDLSCs using microarrays, and miR-874-3p is among the downregulated miRNAs [40]. Here, we found that miR-874-3p was downregulated during the osteogenic differentiation of PDLSCs, and its overexpression inhibited osteogenesis. Bioinformatics algorithms showed that FER1L4 contains a miR-874-3p binding site, and their direct interaction was confirmed by luciferase reporter assays. Further, miR-874-3p reversed the stimulatory effects of FER1L4 on osteogenic differentiation, indicating the involvement of the FER1L4/miR-874-3p regulatory network during the osteogenic differentiation of PDLSCs. However, FER1L4 also absorbs other miRNAs, such as miR-106a-5p [26, 29], miR-18a-5p [30], and miR-372 [31], other miRNAs and their target genes may also participate in the mechanism underlying the regulatory effects of FER1L4.

FER1L4/miR-874-3p regulated osteogenic differentiation via targeting VEGFA. Analyses of TargetScan and starBase predicted that the 3′-UTR of the VEGFA mRNA contains a putative miR-874-3p binding site. A recent study using bioinformatics also suggested that miR-874-3p binds to VEGFA mRNA [40]. In the current study, the binding of miR-874-3p to VEGFA was confirmed by luciferase reporter assays. However, another study showed that miR-874 directly targets STAT3, leading to the inhibition of the STAT3 pathway and downregulation of VEGFA [41]. Thus, other factors or pathways may also be involved in the FER1L4/miR-874-3p/VEGFA regulatory network. VEGFA, an angiogenic/vasculogenic factor, is an enhancer of angiogenesis and osteogenesis. Treatment of PDLSCs with VEGFA increases the accumulation of calcium nodules and the formation of bone tissue [42]. Thus, FER1L4 can upregulate VEGFA expression by absorbing miR-874-3p to participate in the osteogenesis of PDLSCs.

## Conclusion

**Fig. 8:**
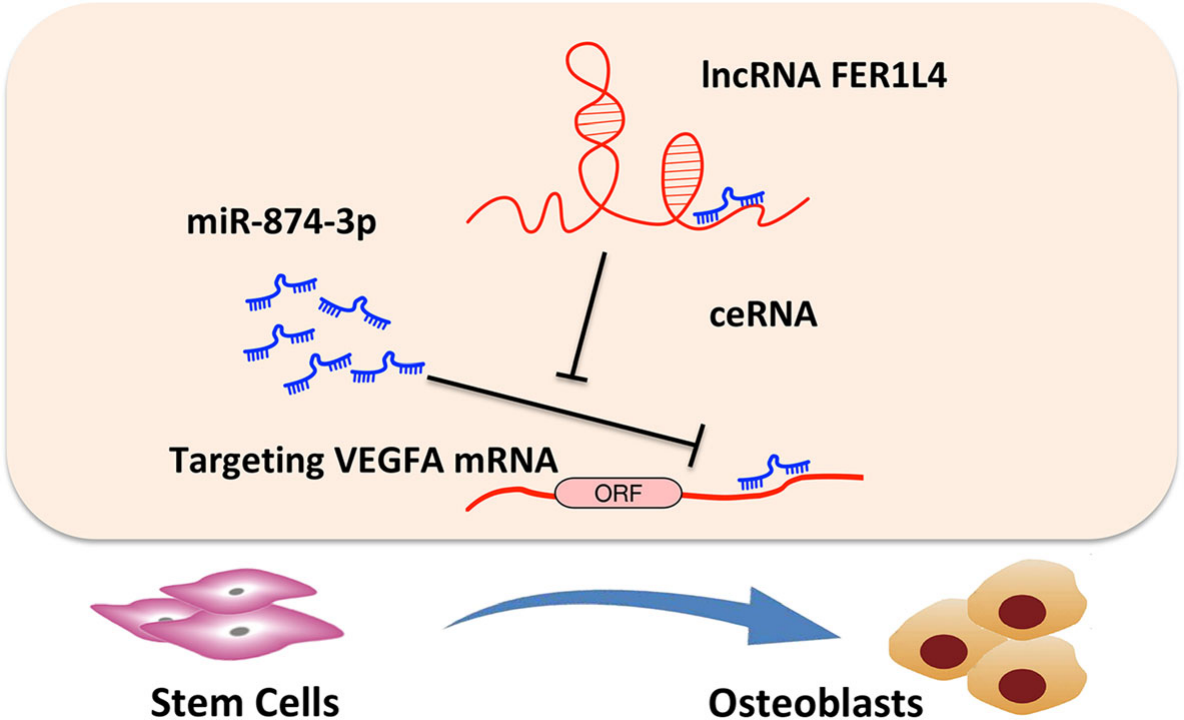
Schematic diagram showing the regulation of osteogenesis by FER1L4

FER1L4 positively regulates the osteogenic differentiation of PDLSCs. Mechanistically, FER1L4 absorbs miR-874-3p, which directly regulates VEGFA, a crucial gene in osteogenic differentiation (Fig. 8). This regulatory network may lead to the development of new approaches to promote the osteogenic potential of PDLSCs and periodontal regeneration.

## Supplementary information

**Additional file 1: Figure S1.** pQLL-FER1L4 vector and transfection efficiency. (A) Schematic diagram illustrating the pQLL-FER1L4 vector. (B) Transfection efficiency of green fluorescent protein (GFP) in PDLSCs. (C) Relative FER1L4 expression in the FER1L4 overexpression and knockdown groups. **Table S1.** Sequences of RNA and DNA Oligonucleotides.

## Data Availability

The datasets used during the current study are available from the corresponding authors on reasonable request.

## Abbreviations

ALP: Alkaline phosphatase
BCA: Bicinchoninic acid
CT: Computed tomography
FER1L4: Fer-1-like family member 4
GAPDH: Glyceraldehyde 3-phosphate dehydrogenase
H&E: Hematoxylin and eosin
lncRNA: Long non-coding RNA
miRNA: MicroRNA
miR-NC: MicroRNA control
mRNA: Messenger RNA
MU: Mutant
ncRNA: Non-coding RNA
OCN: Osteocalcin
PDLSC: Periodontal ligament stromal cell
PLGA: Polylactic-co-glycolic acid
qRT-PCR: Quantitative reverse transcription polymerase chain reaction
RUNX2: Runt-related transcription 2
siFER1L4: Small interfering RNAs targeting FER1L4
siNC: Small interfering RNA control
siRNA: Small interfering RNA
UTR: Untranslated region
VEGFA: Vascular endothelial growth factor A
WT: Wild-type
