# Supplementary material for: Long non-coding RNA FER1L4 promotes osteogenic differentiation of human periodontal ligament stromal cells via miR-874-3p and vascular endothelial growth factor A
Source: Stem Cell Res Ther. 2020 Jan 3;11:5. doi: 10.1186/s13287-019-1519-z (PMC6942378; doi:10.1186/s13287-019-1519-z)
Supplement: Supplementary file 1 — Additional file 1: Figure S1. pQLL-FER1L4 vector and transfection efficiency. (A) Schematic diagram illustrating the pQLL-FER1L4 vector. (B) Transfection efficiency of green fluorescent protein (GFP) in PDLSCs. (C) Relative FER1L4 expression in the FER1L4 overexpression and knockdown groups. Table S1. Sequences of RNA and DNA Oligonucleotides. [file 13287_2019_1519_MOESM1_ESM.pdf]

Supplementary Figure S1

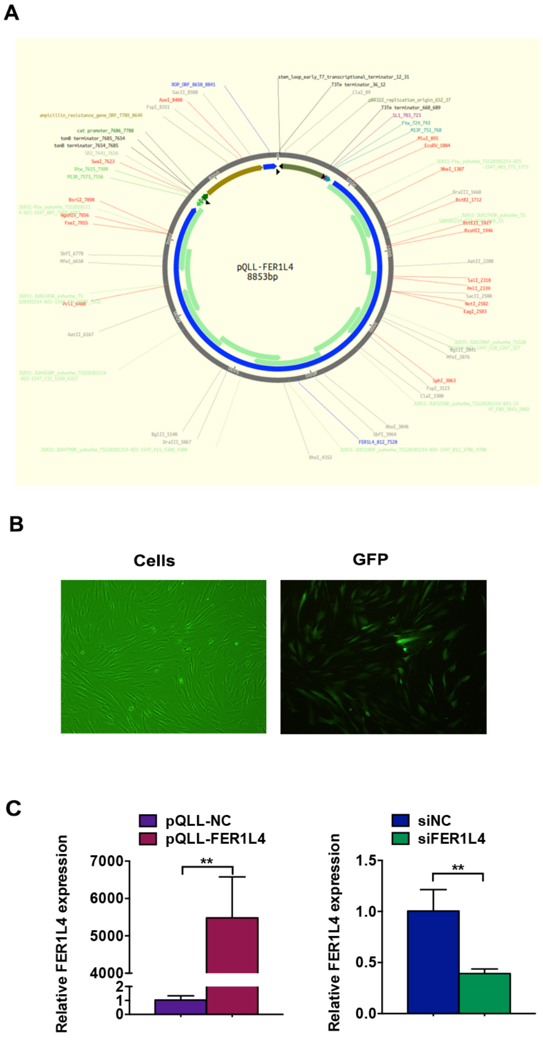

Figure S1. pQLL-FER1L4 vector and transfection efficiency. (A) Schematic diagram illustrating the pQLL-FER1L4 vector. (B) Transfection efficiency of green fluorescent protein (GFP) in PDLSCs. (C) Relative FER1L4 expression in the FER1L4 overexpression and knockdown groups.

Supplementary Table 1. Sequences of RNA and DNA Oligonucleotides

Name	Sense Strand/Sense Primer (5'-3')	Antisense Strand/Antisense Primer (5'-3')
**Primers for qRT-PCR**		
FER1L4	CCGTGTTGAGGTGCTGTTC	GGCAAGTCCACTGTCAGATG
ALP	ATGGGATGGGTGTCTCCACA	CCACGAAGGGGAACTTGTC
RUNX2	CCGCCTCAGTGATTTAGGGC	GGGTCTGTAATCTGACTCTGTCC
OCN	CACTCCTCGCCCTATTGGC	CCCTCCTGCTTGGACACAAAG
VEGFA	AAGGAGGAGGGCAGAATCAT	CCAGGCCCTCGTCATTG
GAPDH	GGTCACCAGGGCTGCTTTTA	GGATCTCGCTCCTGGAAGATG
**siRNAs and miRNA mimics**		
siFER1L4	CAGGACAGCUUCGAGUUAATT	UUAACUCGAAGCUGUCCUGTT
siNC	UUCUCCGAACGUGUCACGUTT	ACGUGACACGUUCGGAGAATT
miR-874-3p	CUGCCCUGGCCCGAGGGACCGA	GGUCCCUCGGGCCAGGGCAGUU
miR-NC	UCACAACCUCCUAGAAAGAGUAGA	UACUCUUUCUAGGAGGUUGUGAUU
